# Intrinsic Toxicity of Unchecked Heterochromatin Spread Is Suppressed by Redundant Chromatin Boundary Functions in *Schizosacchromyces pombe*

**DOI:** 10.1534/g3.115.018663

**Published:** 2015-05-08

**Authors:** Jennifer F. Garcia, Bassem Al-Sady, Hiten D. Madhani

**Affiliations:** Department of Biochemistry and Biophysics, University of California, San Francisco, California 94158

**Keywords:** Clr4, Epe1, boundary, fission yeast, heterochromatin

## Abstract

Effective boundary mechanisms halt the spread of repressive histone methylation. In the fission yeast *Schizosacchromyces pombe*, two factors/elements required for boundary function have been described, the jmjC protein Epe1 and binding sites for the RNA polymerase III transcription factor TFIIIC. Perplexingly, individual mutation of Epe1 or TFIIIC sites produces only mild boundary defects, and no other boundary factors have been identified. To approach these issues, we developed a synthetic reporter gene tool that uses a tethered Clr4 histone H3K9 methyltransferase and monitors the ability of a DNA element to block heterochromatin spread. The inverted repeat (*IR*) that flanks the *mat2/3* silent mating-type cassette region demonstrates strong boundary activity compared to sequences that flank pericentromeric heterochromatic repeats. Rather than acting in the same inhibitory pathway, Epe1 and TFIIIC sites mediate boundary function of the *IR* via the two parallel and largely redundant pathways. We also use the system to demonstrate that HP1/Swi6 promotes boundary activity in addition to promoting silencing and acts in the same pathway as Epe1. Inhibition of heterochromatin spread at the endogenous *IR* element also requires either Epe1 or TFIIIC sites. Strikingly, mutation of both mechanisms results in growth inhibition that is associated with the spread of heterochromatin over many kilobases to the nearest essential gene and the near-complete silencing of several intervening euchromatic genes. The growth defect is reversed by deletion of *clr4*+, indicating that the redundant boundary mechanisms protect cells from intrinsic toxicity caused by the spread of heterochromatin.

The lateral spread of repressive histone H3 lysine 9 (H3K9) methylation occurs from yeast to humans and is counteracted by action of DNA elements called boundary elements (also termed barriers). Boundaries been implicated in important cellular events, including differentiation (Handoko *et al.* 2011), genome imprinting (Chao *et al.* 2002), and cancer (Ceol *et al.* 2011; Fazi *et al.* 2007). Despite the prevalence of chromatin boundaries in humans (Wang *et al.* 2012) and other organisms (Srinivasan and Mishra 2012) and their biological importance, their detailed mechanism of action is not well-understood in any system. Herein, we refer to “boundary elements” as sequences that have been functionally validated to limit heterochromatin spread and “boundary-associated sequences” as sequences present at boundaries that may or may not have been shown functionally to have a role in limiting heterochromatin spread. An important class of boundary elements germane to this study is binding sites for the RNA Polymerase III (RNAPIII) general transcription factor TFIIIC. First identified in a transfer RNA (tRNA) gene that flanks the *HMR***a** silent mating type cassette in *S. cerevisiae* by Donze and Kamakaka (2001) and Donze *et al.* (1999), these are now widely recognized to serve as boundary element in a wide variety of eukaryotes (Kirkland *et al.* 2013).

In the fission yeast *Schizosacchromyces pombe*, three classes of boundary-associated sequences have been described: the *tRNA* clusters and *IRC* elements that flank pericentromeric heterochromatin domains, and the inverted repeat (*IR*) elements of the mating type locus (Cam *et al.* 2005; Noma *et al.* 2001; Partridge *et al.* 2000; Scott *et al.* 2006). Two factors are enriched at boundary sequences, TFIIIC and Epe1. TFIIIC-binding sites have been reported to promote boundary activity of both the tRNA gene cluster of the inner centromere and of the *IR* element (Noma *et al.* 2006). The *IR* element and tRNA genes contain binding sites for TFIIIC termed *B-box*es that are important to boundary activity (Noma *et al.* 2006). At the *IR* element, however, the action of the TFIIIC sites was evident only when the HP1 protein Swi6 was overexpressed via the introduction of three copies of the *swi6*+ gene (Noma *et al.* 2006). At the tRNA gene cluster, the recruitment of TFIIIC correlates with the recruitment of RNAPIII and tRNA transcription (Noma *et al.* 2006). Although the *IR* elements do not recruit RNAPIII, transcription of the *IR* element still occurs (Noma *et al.* 2006). Whether the transcription of either sequence is necessary for boundary activity is unclear. The *IRC* sequence, in contrast, does not recruit TFIIIC (Noma *et al.* 2006). Like boundary factors in higher organisms (Gerasimova *et al.* 2000), TFIIIC in *S. pombe* has been implicated in promoting high-order nuclear organization of the DNA (Noma *et al.* 2006).

Epe1 is a jmjC domain−containing protein required in efficient boundary activity in *S. pombe* (Ayoub *et al.* 2003) and antagonizes transcriptional silencing within heterochromatin (Trewick *et al.* 2005). Although it is related to histone demethylases, Epe1 has yet to be shown to have this activity. Paradoxically, the HP1 protein that promotes heterochromatin spread, Swi6, recruits Epe1 to heterochromatin (Zofall and Grewal 2006). This potentially problematic recruitment mechanism is modulated by a ubiquitin-dependent mechanism to avoid the disruption of silencing. Specifically, Epe1 is actively degraded from heterochromatic domains by an E3 ligase, thus leaving Epe1 enriched at the boundary sequences (Braun *et al.* 2011). What function Epe1 plays at boundaries, however, remains unknown.

We report here the development of a reporter gene system and its use to assess and analyze the potential of DNA sequences to encode heterochromatin boundaries in fission yeast. We observe that both the tRNA gene cluster from *cen1*+ and the *IR* element that flanks the left side of the silent mating type locus (*IR-L*) exhibit boundary activity. We find that the *IR-L* element is affected minimally by mutations that remove Epe1 and or *B-box* elements, but the removal of both results in the spread of silencing. We also use the system to show that Swi6 is required for limiting heterochromatin spread. Although validating that the endogenous *IR* element requires the two redundant pathways to promote boundary activity, we discovered that the function of a single boundary is essential for normal cell growth. Specifically, cells harboring deletion of *B-box*es in the right *IR* repeat and an *epe1*∆ mutation display the ectopic spread of heterochromatin that reaches the first nearest essential gene and dramatically slowed growth. The latter is reversed by deletion of *clr4*+. These observations indicate that redundant pathways promote boundary functions to ensure proper cell growth by suppressing the intrinsic toxicity of heterochromatin spread that can be produced from the inactivation of a single boundary.

## Materials and Methods

### Strain construction

Strains carrying the reporters targeted to the *can1*+ locus (PM1402, 1485, 1517, 1508, 1550, 1591, 1572, 1779) were constructed by transforming the corresponding reporter construct into PM04. Then the strains expect for PM1402 and PM1485 were crossed to an *h*+ strain containing *clr4Δ*::*hph1MX-Gal4DBD-clr4-CDΔ* and dissected for *h−* isolates carrying both the reporter and the Clr4-tethering construct. To generate PM1860 and PM1809, an *epe1Δ*::*kanMX* construct was transformed into PM1779 and PM1572, respectively. PM1863 and PM1800 were obtained by transforming the *swi6Δ*::*kanMX* construct into PM1572 and PM1779, respectively. A *swi6Δ*::*leu1+* construct was transformed into PM1860 to produce PM1925. To create reporter strains to monitor heterochromatin spread from the mating type locus, we used the following primers to amplify the *ura4*+ reporter and flanking targeting homology from SPK787 (Grewal lab): 5′-CTCGTTTAGTCGCAATCTACAC-3′ and 5′-CTGTAGTAGTCGTCTGAAGATTGC-3′. This polymerase chain reaction (PCR) fragment was then transformed into wild-type, *epe1Δ*::*kanMX*, or *epe1Δ*::*kanMX clr4Δ*::*hph1MX h*- yeast strains to generate PM2000, 2004, or 2031, respectively. PM1996 was obtained by crossing and dissecting SPK787 (Grewal lab) to an *h*+ strain. PM1996 is an *h*− isolate that contained the mutated IR-R with the *ura4*+ reporter and did not overexpress swi6 that grew on SC-Ura and failed to grow on SC-Leu. PM2008 was produced by transforming an *epe1Δ*::*kanMX* construct into PM1996. Strains are listed in Table 1.

**Table 1:** Yeast strains used in this study

Strain	Genotype
PM0004	*h- ade6-M210*, *leu1-32*, *ura4-D18*, *smt0*
PM1402	*h- can1*::*ura4+-ade6+*, *ade6-M210*, *leu1-32*, *ura4-D18*, *smt0*
PM1485	*h- can1*::*ura4+-4xGal UAS-ade6+*, *ade6-M210*, *leu1-32*, *ura4-D18*, *smt0*
PM1517	*h- can1*::*ura4+-ade6+*, *clr4∆*::*hphMX-Gal4DBD-clr4-CD∆*, *ade6-M210*, *leu1-32*, *ura4-D18*, *smt0*
PM1508	*h- can1*::*ura4+-4xGal UAS-ade6+*, *clr4∆*::*hphMX-Gal4DBD-clr4-CD∆*, *ade6-M210*, *leu1-32*, *ura4-D18*, *smt0*
PM1550	*h- can1*::*ura4+-IRC-4xGal UAS-ade6+*, *clr4∆*::*hphMX-Gal4DBD-clr4-CD∆*, *ade6-M210*, *leu1-32*, *ura4-D18*, *smt0*
PM1591	*h- can1*::*ura4+-tRNA-4xGal UAS-ade6+*, *clr4∆*::*hphMX-Gal4DBD-clr4-CD∆*, *ade6-M210*, *leu1-32*, *ura4-D18*, *smt0*
PM1572	*h- can1*::*ura4+-IR-L-4xGal UAS-ade6+*, *clr4∆*::*hphMX-Gal4DBD-clr4-CD∆*, *ade6-M210*, *leu1-32*, *ura4-D18*, *smt0*
PM1779	*h- can1*::*ura4+-IR-L MT1 (-327bp)-4xGal UAS-ade6+*, *clr4∆*::*hphMX-Gal4DBD-clr4-CD∆*, *ade6-M210*, *leu1-32*, *ura4-D18*, *smt0*
PM1809	*h- can1*::*ura4+-IR-L MT1 (-327bp)-4xGal UAS-ade6+*, *clr4∆:hxphMX-Gal4DBD-clr4-CD∆*, *epe1∆*::*kanMX*, *ade6-M210*, *leu1-32*, *ura4-D18*, *smt0*
PM1860	*h- can1*::*ura4+-IR-L-4xGal UAS-ade6+*, *clr4∆*::*hphMX-Gal4DBD-clr4-CD∆*, *epe1∆*::*kanMX*, *ade6-M210*, *leu1-32*, *ura4-D18*, *smt0*
PM1863	*h- can1*::*ura4+-IR-L-4xGal UAS-ade6+*, *clr4∆*::*hphMX-Gal4DBD-clr4-CD∆*, *swi6∆*::*kanMX*, *ade6-M210*, *leu1-32*, *ura4-D18*, *smt0*
PM1800	*h- can1*::*ura4+-IR-L MT1 (-327bp)-4xGal UAS-ade6+*, *clr4∆*::*hphMX-Gal4DBD-clr4-CD∆*, *swi6∆*::*kanMX*, *ade6-M210*, *leu1-32*, *ura4-D18*, *smt0*
PM1925	*h- can1*::*ura4+-IR-L-4xGal UAS-ade6+*, *clr4∆*:: *hphMX -Gal4DBD-clr4-CD∆*, *epe1∆*::*kanMX*, *swi6∆*::*leu1+*, *ade6-M210*, *leu1-32*, *ura4-D18*, *smt0*
PM1996	*h- IR-R*::*IR-R-B-boxes∆-ura4+*, *ade6-M210*, *leu1-32*, *ura4-D18*, *smt0*
PM2000	*h- IR-R*::*IR-R-ura4+*, *ade6-M210*, *leu1-32*, *ura4-D18*, *smt0*
PM2004	*h- IR-R*::*IR-R-ura4+*, *epe1∆*::*kanMX*, *ade6-M210*, *leu1-32*, *ura4-D18*, *smt0*
PM2008	*h- IR-R*::*IR-R-B-boxes∆-ura4+*, *epe1∆*::*kanMX*, *ade6-M210*, *leu1-32*, *ura4-D18*, *smt0*
PM2031	*h- IR-R*::*IR-R-B-boxes∆-ura4+*, *epe1∆*::*kanMX*, *clr4∆*::*hphMX*, *ade6-M210*, *leu1-32*, *ura4-D18*, *smt0*

### Silencing plate assays

Cultures were grown overnight at 30° in 1× rich media (YS) to saturation. Cultures were then back diluted to OD_600_ = 0.15 and grown at 30° till the reached an OD_600_ ∼0.6−0.8. Cultures were then back diluted to OD_600_ = 0.6. From this seven 1:5 serial dilutions were made. Dilutions were pinned onto YS, YS + 5-FOA (1 mg/mL 5-FOA; Research Products International), SC, or PMG + CAN (0.6 mg/mL canavanine; Sigma-Aldrich) plates.

### Chromatin immunoprecipitation analysis of H3K9Me

Chromatin immunoprecipitation assays were performed as described in Rougemaille *et al.* 2008 with the following changes: once cultures reach an OD600 0.8−1.0, 40−80 ODs of cells were crosslinked for 20 min by adding formaldehyde (final concentration = 1%) whereas cultures were shaking at 30°. Cells were lysed by beadbeating with Zirconia beads (seven 1-min full-power cycles with a 2-min rest on ice). The chromatin fraction was sonicated using Diagenode Bioruptor water bath sonicator for two 15-min cycles (power: high, 30 sec on, 60 sec off). Sonicated whole-cell extract from approximately 15−20 ODs of cells were used to do three replicate IPs using 2 µL of anti-H3k9Me2 (Ab1220; Abcam) for each IP. Antibody bound protein/DNA was purified using Protein A dynabeads. DNA from IP and WCE was quantified by qPCR. The primer sets used can be found in Table 2.

**Table 2:** qPCR primers used in this study

qPCR Target	Primer Name	Sequence
*ura4+*	P581	5′ - CAG CAA TAT CGT ACT CCT GAA - 3′
	P582	5′ - ATG CTG AGA AAG TCT TTG CTG - 3′
*act1+*	P86	5′ - CAA CCC TCA GCT TTG GGT CTT G - 3′
	P87	5′ - TCC TTT TGC ATA CGA TCG GCA ATA C - 3′
Mating type locus	P1650	5′ - TGA CGT AAT TTG AAG TAC AAA AGG A - 3′
	P1651	5′ - CGG CCT GTA AAG TAT CAG GAG T - 3′
*rga7+ mRNA*	rga7_p2_for	5′ - GAC GCA GGT AAC GTT GAA GAC - 3′
	rga7_p2_rev	5′ - CAA GAT GTA TGG TTA AAT GAC GAA TG - 3′
5′ intergenic region of *aim27*	EuChr2_For	5′ - CAA CGT GAG ACA TGT TAA ATC TC - 3′
	EuChr2_Rev	5′ - GGA TTA GGA CAA ACA GTT TGG - 3′
*SPBC1711.04*	EuChr4_For	5′ - GCG CGA AGG TCT ACT CTG TT - 3′
	EuChr4_Rev	5′ - GCA GAA GGG ACA CCA CAA AT - 3′
*SPBC1711.04 mRNA*	1711.04_For1	5′ - CGC GGC GTC TCT CAA GGA AC- 3′
	1711.04_Rev1	5′ - GCT GAT ACG AAG TAA GGC GAA TGA G- 3′
*SPBC1711.05 mRNA*	1711.05_For1	5′ - CTC TTC CTC ATC TGA TTC AGA TAG - 3′
	1711.05_Rev1	5′ - CGT CTT CGT AGT CCG AGA AG - 3′
*SPBC1711.06*	P2705	5′ - TGC TCT TGC TCG TAT TCC TC - 3′
	P2706	5′ - GAA CAT ACG ACC GCT ACG AC - 3′
*rrb1+*	EuChr8_For	5′ - GTC AGC TCT TAC CGT CAA TG - 3′
	EuChr8_Rev	5′ - CTG TAT ATA GGA GCA CGG TGC - 3′
3′ intergenic region of *rrb1+*	EuChr9_For	5′ - AGG CAT TGG ACT TCA AAG GA - 3′
	EuChr9_Rev	5′ - TTT TGC GCA TAG AGA CAT CG - 3′

qPCR, quantitative polymerase chain reaction.

### Plate growth assay

PM2004, PM2008, and PM2031 were grown from frozen stocks on YS plates and grown for 2 d at 30°. Each strain was then streaked for singles onto the same YS plate and grown until single colonies appeared.

### RNA extraction and reverse-transcription quantitative polymerase chain reaction

A total of 6−8 OD600 units of cells were harvested from log phase cultures. Cell pellets were resuspended in 1 mL of Trizol (Ambion). A total of 250 μL of volume of zirocnia beads were added to the resuspension and then vortexed twice for 2.5 min on a cell disruptor. The lysate was cleared then extracted against chloroform. The aqueous layer recovered, after a 10-min incubation at room temperature and 10-min spin, and extracted against of chloroform. After a 10-min spin, the aqueous phase was extracted to a new tube. Isopropanol was added to precipitate the RNA. The RNA pellet was spun down and washed with 75% ethanol. The pellet was then air dried and resuspended in diethylpyrocarbonate H_2_O. Then, 18 μg of RNA was resuspended to a final volume of 45 μL then treated with the Turbo DNase kit (Ambion). A total of 3 µg of DNase-treated RNA was added to 500 ng of oligo dT and 500 ng of random 9-mers and 10 mmol of dNTPs in a final volume of 13 μL and incubated at 65° for 5 min. This mixture was then cooled on ice for 5 min. A final concentration of 1× First Strand buffer, 5 mM DTT, 0.2 μL of Superscript III RT and diethylpyrocarbonate H_2_O were added the RNA primer mix to a final reaction volume of 20 μL. The reaction was incubated at 25° for 5 min, 50° for 1 hr then 70° for 15 min. The resulting cDNA was analyzed in 15-µL quantitative polymerase chain reactions in three technical replicates using primers listed in Table 2.

## Results

### Reporter gene system

**Figure 1:**
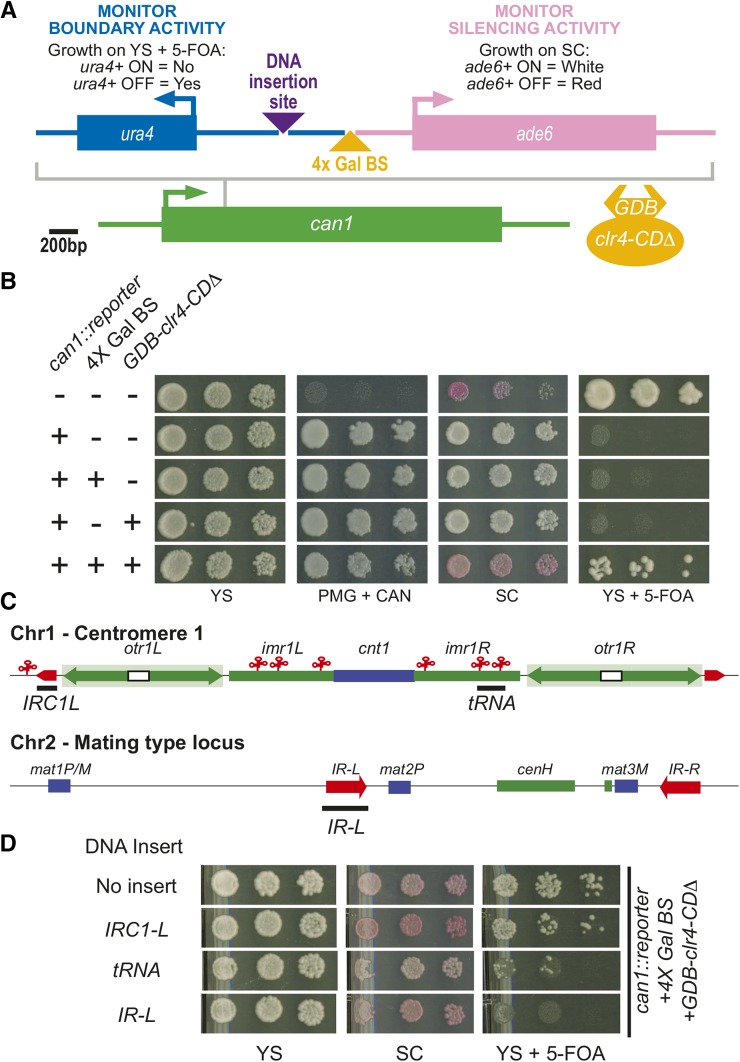
The transfer RNA gene cluster and *IR-L* promote boundary activity in a synthetic boundary reporter. (A) Scaled schematic depicting the setup of the reporter construct that was inserted into the *can1+* locus. Arrows depict the direction of transcription. Purple triangle represents the DNA insertion site for boundary elements to be tested. The yellow triangle indicates the site where the 4× Gal binding sites are inserted, allowing the recruitment of GBD-Clr4-CDΔ. (B) Plate growth assays on various media. Yeast are pinned onto plates with fivefold dilutions from OD_600_ = 0.6 culture. YS, rich media; SC, low adenine media (reads out the expression of the *ade6+* reporter gene); YS + 5-FOA, rich media containing the drug 5-FOA (assesses expression of the *ura4+* reporter gene); PMG + CAN, a minimal media that contains the drug canavanine (reads out the presence of the reporter in the *can1*+ locus). “+” denotes the presence of a construct in the strain analyzed. (C) A schematic depicting two major regions of *S. pombe* heterochromatin, centromere 1 and silent mating type locus on chromosome 2. Red elements depict known boundary elements. Blue elements represent genes while green elements indicate heterochromatic sequences. The black bars describe the regions of the DNA analyzed. (D) Plating growth assays of the reporter strain with three different boundary elements (depicted by black bars in C) inserted into the DNA insertion site with the heterochromatin facing side of the boundary element adjacent to the 4× Gal BS.

To study boundary activity, we created a reporter system to assess boundary activities via simple plate assays. To achieve this we triggered heterochromatin formation ectopically by using a previously described strategy in which the Gal4 DNA-binding domain (GDB) is fused to a version of the H3K9 histone methyltransferase Clr4 that lacks its chromodomain and then recruited to DNA via Gal4 binding sites (Kagansky *et al.* 2009). To read out both boundary and silencing activity of the reporter, we used two reporter genes, *ura4*+ and *ade6*+, which are assayed by sensitivity to the 5-FOA and colony color, respectively (Forsburg 2001). These two reporter genes were inserted the into the *can1*+ locus in a divergent orientation (Figure 1A). Between the *ura4*+ and *ade6*+ reporter genes, we inserted two unique restriction endonuclease sites (Figure 1A). Into the site upstream of the *ade6*+ gene, we inserted four tandem Gal4 UAS binding sites (4× Gal BS) used to tether Clr4 to the reporter. The second restriction endonuclease site was engineered upstream from the 4× Gal BS where DNA elements can be inserted to test for boundary activity.

The parent strain contains inactive allele of *ade6*+ and the deletion allele of *ura4+*. This strain displays growth on YS+5-FOA medium, appears red on low adenine media (SC), and fails to grown on minimal media containing canavanine (PMG+CAN) (Figure 1B). As expected, on insertion of the reporter into the *can1*+ locus, the strain is no longer sensitive to canavanine as is it able to grow on PMG + CAN media (Figure 1B) whereas the *ade6*+ and *ura4*+ reporter genes are expressed at the *can1*+ locus, resulting in white colonies on SC media and a lack of growth on YS+5-FOA medium, respectively (Figure 1B). Both insertion of the 4× Gal BS element and expression of *GDB-clr4-CD∆* yielded silencing of both reporter genes (Figure 1B). H3K9 methylation is induced over the *ura4*+ reporter gene in this strain (supporting information, Figure S1).

### *cen1*+ tRNA clusters and the *IR* element from the *mat2/3* locus confer boundary activity

To test whether our reporter can monitor boundary activity, boundary-associated sequences were placed into the reporter 400 bp upstream from the Clr4-tethering site and 577 bp downstream from the start codon of the *ura4*+ reporter gene. Three endogenous boundary elements were inserted into the reporter with the heterochromatic end of the boundary element adjacent to the 4×Gal BS (Figure 1C, black bars). When a 1.1-kb DNA fragment containing the *IRC* sequence element from the left boundary of *cen1*+ was placed into the reporter construct, there was no change in the amount of growth on YS+5-FOA compared with the reporter without an insert (Figure 1D), indicating that *IRC* element does not promote boundary function in this system. Next, we tested a DNA element from the *cen1*+ that contains an alanine and a glycine tRNA gene that was shown previously to be sufficient to confer boundary activity (Scott *et al.* 2006). This insertion in the reporter reduced growth on YS+5-FOA (Figure 1D), suggesting that the tRNA gene cluster promotes measurable but weak boundary. In contrast, a DNA fragment corresponding to the *IR-L* element induced a strong loss in growth on YS+5-FOA (Figure 1D). The *ade6*+ reporter gene remains silenced as the strain displays a red colony phenotype on SC media (Figure 1D). We observed a reduction in H3K9me over the *ura4*+ reporter gene compared with no DNA insert (Figure S1); however, this reduction was quantitatively modest, indicating that the effectiveness of the boundary is not absolute in this context, at least when the *ura4*+ reporter genes is closely juxtaposed to the site of silencing initiation. Nonetheless, these observations strongly suggest that the *IR-L* element can promote boundary activity. Although the *IR-L* element insert is longer than others tested, data shown below indicate that specific sequences rather than mere length are required for the *IR* element to function.

### Ectopic boundary activity of the *IR-L* element mimics endogenous requirements

**Figure 2:**
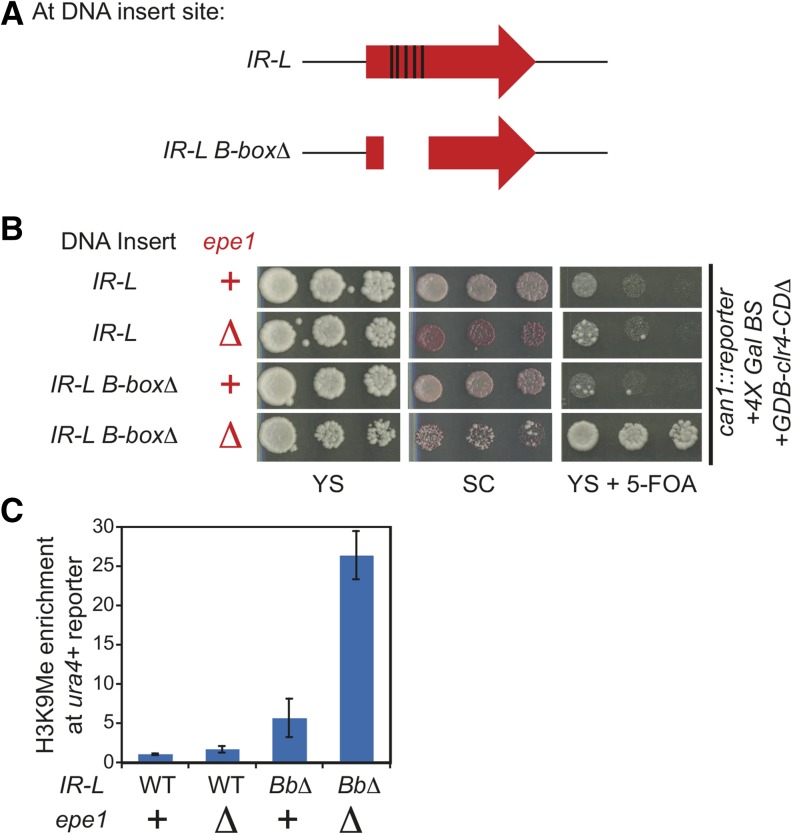
Two parallel pathways govern by Epe1 and TFIIIC mediate the boundary activity of the *IR-L* element. (A) Diagram of the location of the five B-boxes (depicted by black rectangles) found in *IR-L* and an *IR-L* B-box mutant that contains a deletion that spans 327 bp. (B) Plate assays of the wild-type *IR-L*, the *IR-L B-box∆* mutant, *epe1*∆ mutant, or *IR-L B-box*∆ *epe1*∆ double mutant in the reporter. (C) Quantification of the H3K9me chromatin immunoprecipitation enrichment over the *ura4+* reporter gene. IP/WCE values for *ura4+* are normalized to *act1+* IP/WCE signal for that strain. H3K9me levels are then normalized relative to the wild-type *IR-L* boundary reporter strain (column 1). The error bars represent the standard deviation.

To test that the boundary activity we observe with the *IR-L* element in the reporter is functionally related to previously described mechanisms, we examined its requirements for boundary activity. In cells harboring a deletion of *epe1*+, a slight increase of growth on YS+5-FOA (Figure 2B) and a modest 1.7- fold increase in the H3K9me levels over the *ura4*+ gene was observed (Figure 2C). An *epe1∆* mutant also exhibited a red color phenotype on SC plates, diagnostic of increased silencing of *ade6*+. This finding indicates that Epe1 is recruited to the reporter by heterochromatin formation via a tethered Clr4 and can down-regulate H3K9me levels, which is consistent with the known role of heterochromatin in recruiting Epe1 (Braun *et al.* 2011; Zofall and Grewal 2006).

We next tested the requirement of the transcription factor, TFIIIC, by inserting a mutant of the *IR* element that contained a 327-bp deletion spanning the five *B-box* elements that recruit TFIIIC into the reporter strain (Figure 2A). Although the *ade6*+ reporter gene remains repressed, the *IR-L B-box* mutant displays increased growth on YS+5-FOA compared with the wild-type *IR-L* element (Figure 2B). Analogously, a 5.6-fold increase in H3K9me over the *ura4*+ gene was observed (Figure 2C). Thus, *B-box*es and, by inference, TFIIIC are required for full boundary activity of the *IR-L* sequence in the reporter and suggest that the activity observed in our reporter is that of functional boundary formation.

### TFIIIC and Epe1 work in two separate pathways to form a robust boundary activity at *IR-L*

The aforementioned results demonstrate that we have successfully created a reporter gene tool to monitor the boundary activity of the *IR* sequence. Additionally, we find that the reporter mimics endogenous requirements for Epe1 and the B-boxes but that neither factor is absolutely essential for the boundary activity of the *IR* element. These faint phenotypes could be due to the fact that heterochromatin induced in our system is intrinsically too weak to spread over the *IR-L* element or that Epe1 and TFIIIC work in two redundant pathways to promote boundary function. To test the latter, we transformed in *epe1∆*::*kanMX* into the reporter strain containing the *IR-L B-box* deletion. As predicted by the redundancy model, we observed that this double mutant exhibited significantly increased growth on YS+5-FOA (Figure 2B) and a strong increase in the levels of H3K9me levels over the *ura4*+ gene (Figure 2C), indicating that heterochromatin can spread across the *IR-L* element. We conclude that Epe1 and TFIIIC to work in two parallel pathways to promote boundary function of the *IR* elements.

### Swi6 and Epe1 function in the same silencing-inhibitory pathway

**Figure 3:**
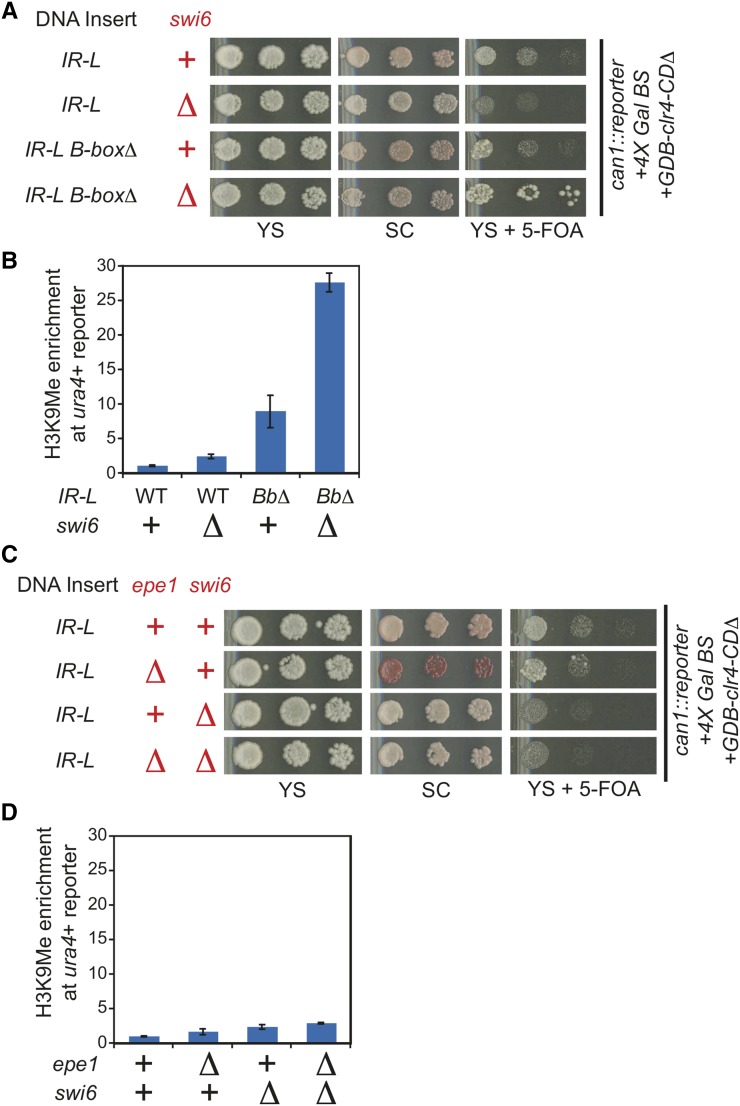
Swi6 promotes boundary activity through the Epe1 pathway. (A) The *swi6∆*::*kanMX* mutation was introduced into reporter strains carrying *IR-L* or the *IR-L B-box*∆ mutation and boundary activity assayed by plate growth. (B) H3K9me chromatin immunoprecipitation enrichment over the *ura4+* reporter gene for the strains assayed in A. Normalization as described in Figure 2C. Error bars represent the standard deviation. (C−D) The *swi6Δ*::*leu1+* mutation was introduced into the reporter strain containing *epe1Δ*::*kanMX* and tested for boundary activity as in A-B. Error bars represent the standard deviation.

If Epe1 and TFIIIC worked in two separate pathways, then factors that function only in one of these two pathways would be anticipated to have distinct phenotypes in the wild type, *epe1*Δ, or the *B-box* deletion strains. We suspected that the HP1 protein, Swi6, is involved in boundary function because it has been shown to recruit Epe1 to heterochromatin and boundary elements. We took advantage of the previously described ability of tethered Clr4 to bypass a requirement for Swi6 promote heterochromatin formation (Kagansky *et al.* 2009). A *swi6∆*::*kanMX* construct was transformed into reporter strains containing either the wild-type *IR-L* or the *IR-L B-box* deletion mutation. As expected from previous work (Kagansky *et al.* 2009), silencing of the *ade6*+ reporter gene is unaffected by deletion of *swi6*+. If Swi6 functions in the Epe1 pathway to limit heterochromatin spread, the same synergistic increase in silencing over the *ura4*+ reporter gene observed in the *epe1*∆ *B-box* double mutant should be observed in a *swi6*∆ *B-box* double mutant. This is what we observed (Figure 3, A and B). The *swi6*∆ single mutant displayed a similar phenotype to an *epe1*Δ mutant in the *IR-L* reporter strain, exhibiting a minor 2.4-fold increase of H3K9me levels compared with the wild type (Figure 3B). These observations support the model that Swi6 and Epe1 affect boundary function through the same pathway and act in parallel to TFIIIC. The amount of repression on the plate assays for the *ura4*+ reporter gene in the *swi6*∆ *B-box* double mutant was not as strong as the repression observed for the *epe1*∆ *B-box* double mutant (Figure 3A). Further, we also noticed that repression of the *ade6* reporter was less efficient in the context of *swi6*∆ when compared *epe1∆*. Both the weaker repression at *ura4* and *ade6* in the *swi6*∆ *B-box* double mutant could be explained if in our reporter system the requirement for Swi6 in heterochromatic silencing is not bypassed fully. To further test whether Swi6 acts in the same pathway as Epe1, we created a double mutant of *epe1*∆ and *swi6*∆ in the WT *IR-L* reporter strain. As expected from a model in which they function in the same genetic pathway, we did not observe a more severe phenotype for the double mutant when compared the single mutants in this reporter strain (Figure 3, C and D).

### Redundant pathways act to prevent toxic heterochromatin spread

**Figure 4:**
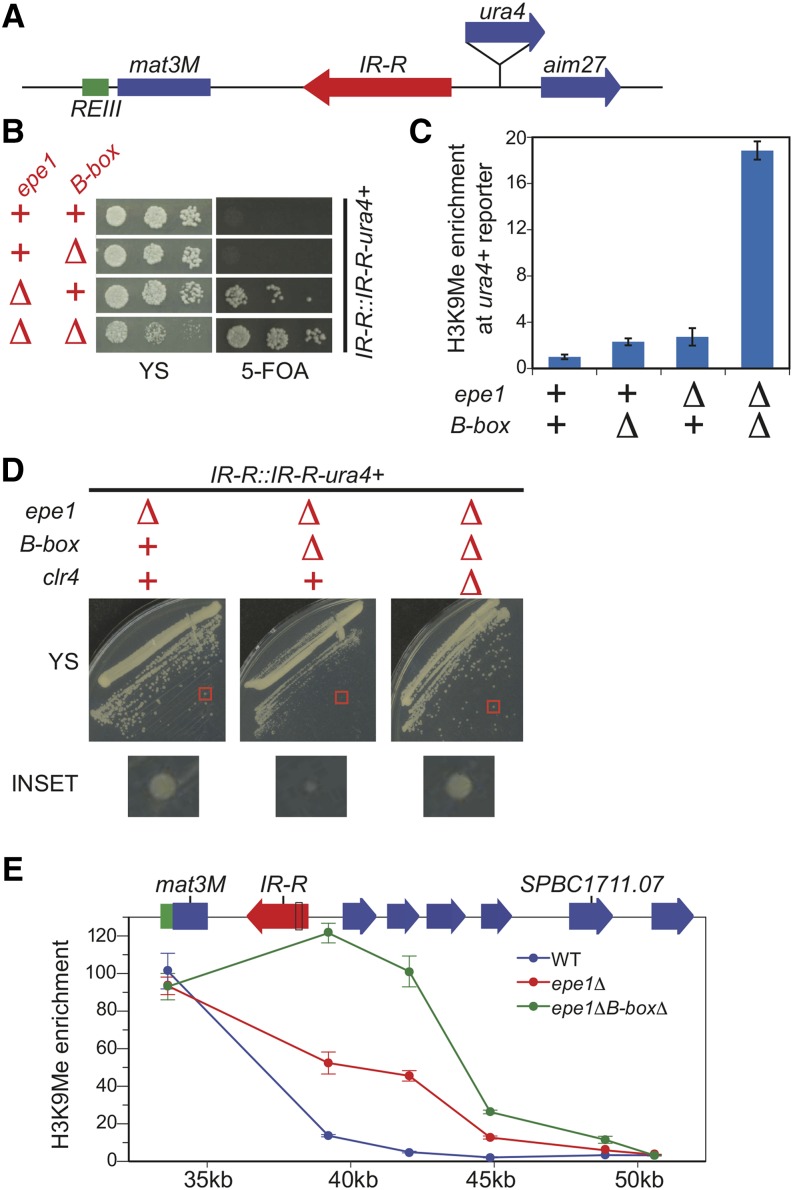
Redundancy in Epe1 and TFIIIC function prevent a toxic spread of heterochromatin. (A) Depiction of the reporter strain used to test the boundary activity of the endogenous *IR* element where the *ura4+* reporter gene is inserted downstream of the *IR-R* element. (B) Plate assay of the wild-type reporter strain and the reporter strain harboring a mutant where B-boxes are deleted in *IR-R*, an *epe1∆*::*kanMX* mutant or both. (C) H3K9me chromatin immunoprecipitation (ChIP) enrichment over the *ura4+* reporter gene for the strains assayed in B. Normalization is the same as described in Figure 2C expect that H3K9me levels are normalized to the wild-type reporter (Column 1). Error bars represent the standard deviation. (D) The endogenous boundary reporter strain containing an *epe1∆*::*kanMX* mutant are grown on the same YS plate to assay their growth after introduction of the *IR-R B-Box* deletion mutation or a *clr4*∆ mutation. The red box highlights a single colony shown in the inset. (E) H3K9me ChIP at heterochromatin and euchromatin regions flanking the *IR-R* boundary in the endogenous boundary reporter strain in the context of either wild-type (WT), the *epe1∆*::*kanMX* (*epe1∆*) single, or *epe1∆* and B-box deletion double mutant (e*pe1∆B-box∆*). X-axis is the distance (in kilobases) from the start of the annotated mating type locus. IP/WCE values for each primer set are normalized to *act1*+ IP/WCE values. Error bars represent the standard deviation.

Our analysis using our synthetic reporter tool suggested that two parallel pathways to promote boundary function of the *IR* element. To test whether this was the case at an endogenous boundary, we used a strain described previously to assay the boundary activity of the *B-box*es elements (Noma *et al.* 2006). In these strains, a *ura4*+ reporter gene was introduced in the euchromatic region downstream of the wild-type *IR-R* element or a mutant *IR*-R element that contained a deletion that spanned the five *B-box*es (Figure 4A). Additionally, *swi6*+ in this strain was overexpressed by the insertion of three copies of the gene into its chromosomal locus (Noma *et al.* 2006). To test whether two parallel pathways mediate the boundary activity of the *IR* element, we replaced the 3X-*swi6*+ allele with a wild-type copy and then introduced *epe1*∆::*kanMX* by transformation.

To observe whether heterochromatin spread is observed past the boundaries, we plated these strains on YS and media containing 5-FOA. As expected, we did not observe any spread of heterochromatin when the *B-box*es of the *IR* element and Epe1 are both present (Figure 4B). In the *B-box∆* mutant background, we did not see any growth on 5-FOA (Figure 4B) and we observed only a small increase of H3K9me levels over the *ura4*+ gene (Figure 4C), indicating that the boundary function of *IR-R* element is not severely compromised when TFIIIC sites are removed. This finding differs from the published result because *swi6*+ is not overexpressed in our strains, resulting in less heterochromatin spread from the mating type locus. An *epe1*∆ mutant exhibits heterochromatin spread from the mating type locus into our *ura4*+ reporter gene as it shows mild growth on 5-FOA (Figure 4B) with a corresponding increase in H3K9 levels over the *ura4*+ reporter gene (Figure 4C).

When we analyzed strains that lacked both the *B-box*es and Epe1, we observed increased heterochromatin spread over the *ura4*+ reporter gene (Figure 4C) and increased synergistic growth on 5-FOA (Figure 4B) as we had expected from our reporter. This observation confirms that Epe1 and TFIIIC act in two redundant pathways to promote full boundary function at endogenous *IR* elements. Strikingly, the double mutant also exhibited a strong growth defect on YS (Figure 4B).

We tested whether this growth defect, which was not observed in our reporter strain, was caused by unchecked heterochromatin spread caused by the lack of both boundary pathways. To examine this possibility, we introduced a *clr4∆*::*hphMX* deletion into the endogenous reporter strain carrying *epe1*∆ and the *B-boxes*∆ mutations. As shown in Figure 4D, the growth in the boundary reporter strain lacking both the *B-box*es and Epe1 is fully restored when *clr4*+ is deleted, indicating that the slow growth defect is caused by heterochromatin formation. The authors of a previous study reported that deletion of the *IR-R* element but did not report a growth defect (Singh and Klar 2002). This result again suggests that Epe1 can have boundary element-independent effects on the spread of heterochromatin and therefore the maintenance of normal cell growth.

**Figure 5:**
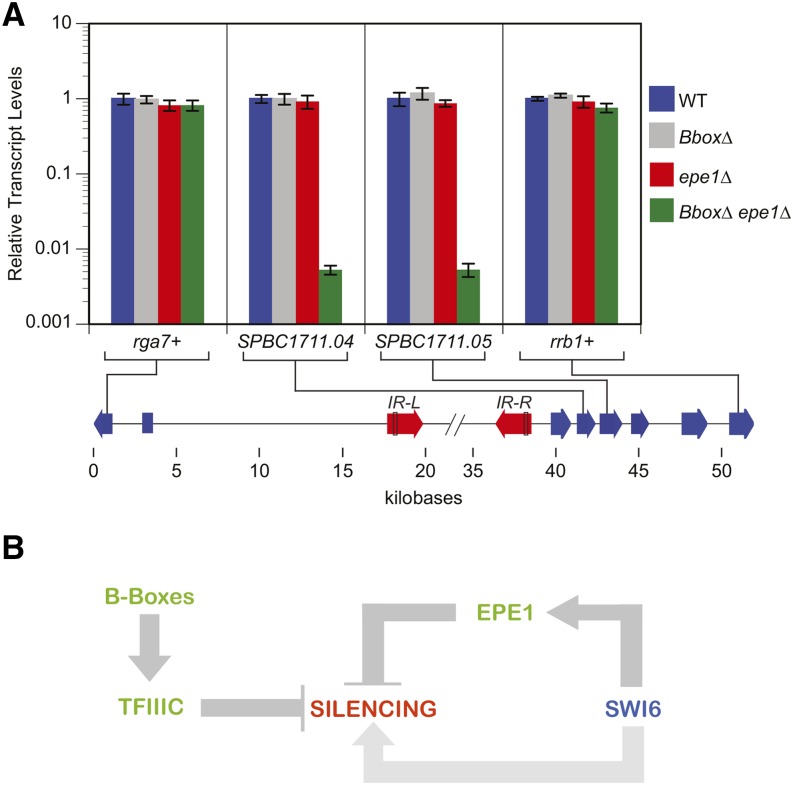
A model depicting the pathways that negatively and positively modulates heterochromatic transcriptional silencing. (A) Relative transcript levels of messenger RNAs located in the euchromatin regions proximal to the mating type locus in strains described in Figure 4B as determined by reverse-transcription quantitative polymerase chain reaction (qPCR). Transcript levels are normalized to *act1*+ transcript levels. The error bars represent the SD of two to three technical replicates of the qPCR. X-axis is the distance (in kilobases) from the start of the annotated mating type locus. (B) Heterochromatin-induced gene silencing is promoted by the action of HP1 proteins like Swi6 whereas its spread is limited to heterochromatic domains by TFIIIC and Epe1. The *IR* element in *S. pombe* uses the TFIIIC recruiting *B-boxes* as well as Epe1 in two parallel and redundant pathways to promote boundary function. Compromising one pathway weakly reduces boundary activity, whereas inactivating both pathways leads to a catastrophic spread of repressive heterochromatin into neighboring DNA domains, and, thus, becoming detrimental to cell growth. Swi6 is required for Epe1 dependent branch of boundary formation, in addition to its role in promoting heterochromatin spread.

We examined H3K9 methylation to test how far heterochromatin spreads without Epe1 and *B-box*−dependent boundary mechanisms. We observe a strong increase in H3K9 methylation 9 kb from the mating type locus, which not observed in the wild type or as strongly in *epe1*∆ strains (Figure 4E). We do not detect H3K9 methylation past the first gene annotated to be essential for viability, *rrb1+*/*SPBC1711.07*. To probe the impact of ectopic heterochromatin on gene expression, we examined transcript levels for *rrb1+/SPBC1711.07* as well as two nonessential genes that lie between *IR-R* and this gene, *SPBC1711.04* and *SPCB1711.05*. Consistent with the viability of the *B-box*∆ *epe1*∆ double mutant, we did not observe silencing of *rrb1*+/*SPBC1711.07*. In contrast, we observed near-complete silencing (190-fold) both *SPBC1711.04* and *SPCB1711.05* (Figure 5A). Importantly, this profound repression was only observed in the double mutant, consistent with genetic redundancy between *B-box* and Epe1-dependent anti-silencing mechanisms. As a control, we examined the transcript levels for the first gene to the left of mating type locus, *rga7+/ SPBC23G7.08c*. No silencing was evident in any genotype tested (Figure 5A).

## Discussion

Boundary elements, although central to defining the extent of heterochromatin, remain among its least understood features. In *S. pombe*, only two factors have been implicated in heterochromatin boundary activity, the jmjC protein Epe1 and the RNAPIII general transcription factor TFIIIC. Neither has a particularly dramatic impact on heterochromatin spread when mutated and thus either appears insufficient to alone fully define boundary activity. Using a reporter system in *S. pombe* in which histone methyltransferase tethering is used to trigger silencing, we have approached these issues. Our studies lead two novel conclusions. First, the two well-defined inhibitors of heterochromatin spread act redundantly in parallel pathways. Second, heterochromatin spread produced by loss of both pathways at an endogenous boundary results in dramatic growth inhibition.

Our studies of *IR* element in the context of a synthetic boundary reporter gene and at its endogenous site demonstrate unequivocally that Epe1 and sites for TFIIIC function in parallel pathways (Figure 5B), explaining the relatively mild phenotypes of boundary factors/elements obtained in past studies. The fact that we observe the same result in these two contexts indicates that the mechanism of recruitment of the histone methyltransferase does not appear to significantly impact how a boundary functions. That is, the initiation and termination events of heterochromatin assembly are not coupled.

Our finding of redundancy in boundary pathways offers a plausible explanation for why few boundary factors have been identified to date. We anticipate that unbiased forward genetic screens using the boundary reporter and strains defective in either the TFIIIC or Epe1 pathway will identify substantial additional components. Such studies should be highly informative because the underlying biochemical mechanisms by which TFIIIC and Epe1 enforce boundary activity in fission yeast remain opaque: 1) although TFIIIC has been suggested to mediate boundary function by promoting a specific nuclear architecture in *S. pombe* (Noma *et al.* 2006), we have identified nucleosome free regions (Garcia *et al.* 2010) that coincide with both tRNA genes and the TFIIIC sites in the *IR* sequence, suggesting the possibility a sufficiently large gap in the nucleosome array could mediate boundary function as suggested previously (Donze and Kamakaka 2001). Direct evidence for either model is currently lacking. 2) Epe1 has a jmjC domain found in demethylases, but whether it is a *bona fide* demethylase and, if so, what its substrates might be, remains unknown.

Our investigation of the endogenous *IR-R* boundary demonstrates that uncontrolled heterochromatin spread is poorly tolerated *in vivo*. In *Drosophila*, chromosome translocations that cause the spread of heterochromatin into euchromatic regions and inactivation of sensitive reporter genes have been described. These mutations trigger heterochromatin spread via a genetic recombination event. In contrast, we are not aware of an example where an inactivation of an endogenous heterochromatin-euchromatin boundary element produces a toxic spread of heterochromatin. Our findings are striking in that inactivation of a single boundary appears to have highly deleterious effects. While there are many important adaptive features of heterochromatin, its ability to spread makes it an obviously double-edged sword. The evolution of two, layered mechanisms to limit heterochromatin spread may reflect the need for cells to have robust mechanisms to limit its potential for deleterious gene repression. Similar redundancy may exist in other systems that involve repressive histone methylation that has the ability to spread.
